# Genetic Variation in the *Staphylococcus aureus* 8325 Strain Lineage Revealed by Whole-Genome Sequencing

**DOI:** 10.1371/journal.pone.0077122

**Published:** 2013-09-30

**Authors:** Kristoffer T. Bæk, Dorte Frees, Adriana Renzoni, Christine Barras, Natalia Rodriguez, Caroline Manzano, William L. Kelley

**Affiliations:** 1 Department of Veterinary Disease Biology, University of Copenhagen, Frederiksberg, Denmark; 2 Service of Infectious Diseases, University Hospital and Medical School of Geneva, Geneva, Switzerland; National Institutes of Health, United States of America

## Abstract

*Staphylococcus aureus* strains of the 8325 lineage, especially 8325-4 and derivatives lacking prophage, have been used extensively for decades of research. We report herein the results of our deep sequence analysis of strain 8325-4. Assignment of sequence variants compared with the reference strain 8325 (NRS77/PS47) required correction of errors in the 8325 reference genome, and reassessment of variation previously attributed to chemical mutagenesis of the restriction-defective RN4220. Using an extensive strain pedigree analysis, we discovered that 8325-4 contains 16 single nucleotide polymorphisms (SNP) arising prior to the construction of RN4220. We identified 5 indels in 8325-4 compared with 8325. Three indels correspond to expected Φ11, 12, 13 excisions, one indel is explained by a sequence assembly artifact, and the final indel (Δ63bp) in the *spa-sarS* intergenic region is common to only a sub-lineage of 8325-4 strains including SH1000. This deletion was found to significantly decrease (75%) steady state *sarS* but not *spa* transcript levels in post-exponential phase. The sub-lineage 8325-4 was also found to harbor 4 additional SNPs. We also found large sequence variation between 8325, 8325-4 and RN4220 in a cluster of repetitive hypothetical proteins (SA0282 homologs) near the Ess secretion cluster. The overall 8325-4 SNP set results in 17 alterations within coding sequences. Remarkably, we discovered that all tested strains of the 8325-4 lineage lack phenol soluble modulin α3 (PSMα3), a virulence determinant implicated in neutrophil chemotaxis, biofilm architecture and surface spreading. Collectively, our results clarify and define the 8325-4 pedigree and reveal clear evidence that mutations existing throughout all branches of this lineage, including the widely used RN6390 and SH1000 strains, could conceivably impact virulence regulation.

## Introduction

*Staphylococcus aureus* is a leading cause of human disease ranging from minor skin infections to life-threatening endocarditis, pneumonia and septicemia [1]. Adequate treatment and infection control are compounded by the world-wide spread of methicillin resistant *Staphylococcus aureus* (MRSA) strains [2]. The pathogenicity of *S. aureus* relies on a wide array of surface-bound and secreted virulence factors that collectively modulate tissue adherence, cytopathology, and immune evasion [3]. The expression of virulence factors in *S. aureus* are controlled by an exceedingly complex network of regulatory elements [4].

Current models for global virulence regulation in *S. aureus* are based on an enormous wealth of studies predominately performed with the laboratory strain 8325-4 (also designated as RN450), or its derivatives, such as the widely used RN6390. Strain 8325-4 was derived by stepwise removal of three resident prophage from the sepsis isolate 8325 (also designated as RN1 or PS47; reviewed in 5). The 8325-4 (RN450) lineage has thus marked much of the history of *S. aureus* research since its published introduction [6].

A decade ago, 8325-derived strains were shown to harbor reduced activity of the alternative stress sigma factor, SigB, resulting from an 11 base pair (bp) deletion in *rsbU* encoding a phosphatase regulating the dissociation of the complex of SigB and anti-sigma factor RsbW [7]. Since high hemolysin and extracellular protease expression observed in 8325-derived strains could be linked to the low activity of SigB, the usefulness of these strains and their derivatives for studying virulence regulation was questioned [8-10]. To address this issue, an *rsbU+* repaired derivative of 8325-4 was constructed (SH1000) that found considerable subsequent use in studies of *S. aureus* virulence regulation [10].

In a similar manner, 8325-derived strains were also found to have a mutation in *tcaR* leading to a prematurely truncated MarR-like DNA binding transcription factor thought to modulate cell wall active drug resistance [11] as well as virulence factor expression through its control of *sarS* [12]. The loss of TcaR is linked to low *sarS* transcription levels and thus 8325 derivatives (HG001-3) have been prepared that restore not only *rsbU*, but also *tcaR* [5].

Another 8325-4 derivative of particular importance is RN4220, which was constructed by nitrosoguanidine (MNNG) mutagenesis to obtain a strain that features an inactivated restriction system permitting its broad application as an intermediate cloning strain. Recently, deep sequencing showed RN4220 to harbor more than 100 single nucleotide polymorphisms (SNPs) and one additional deletion compared to 8325, consistent with chemical mutagenesis, and thus casting considerable doubt on the suitability of this strain for rigorous studies of metabolic or virulence factor regulation [5,13]. A subsequent study, however, found that a number of the reported SNPs were the result of sequencing errors in the published 8325 reference sequence [14]. Studies with RN4220 have thus led to an appreciation of the notion that the use of reference strains, such as 8325, for SNP discovery analysis may require considerable nucleotide corrections of the reference genome. A separate study of SH1000 by the complete genome hybridization technique (CGH) also revealed sequence differences with the 8325 reference genome [15].

In the widely accepted and published pedigree of 8325-4 [5,16] it is generally understood that this strain was obtained by curing 8325 of its resident prophages in two successive cycles of UV irradiation [6], yielding a strain believed to be little different from its 8325 parent. During the course of deep sequencing studies in our own laboratories using 8325-4, we uncovered numerous mutations compared to the 8325 reference strain, some of which were previously attributed to the chemical mutagenesis step leading to RN4220, as well as other mutations not detected by CGH in SH1000.

The present study presents a refined analysis of 8325-4 mutations compared to its parental strain 8325, including additional nucleotide corrections of the 8325 reference genome, showing 20 SNPs to be present in our deep sequenced strain. A broad analysis of the SNP distribution was undertaken using a sample of 8325 and 8325-4 strains collected worldwide, or obtained from the Network on Antimicrobial Resistance in *Staphylococcus aureus* (NARSA, www.narsa.net). We provide a detailed description of the mutations arising concurrently with prophage removal, and uncover evidence for the existence of an 8325-4 sub-lineage, which includes the widely used *rsbU*+ restored strain SH1000. Of particular note, this sub-lineage harbors a 63 bp deletion in the *spa-sarS* intergenic region which is shown to significantly affect *sarS* mRNA levels. Collectively, these results help to pinpoint steps in the pedigree of strains widely used in *S. aureus* research, modify the 8325 sequence database, and lend caution to genetic manipulation of 8325-4 strains with the knowledge that mutations in this lineage have already or could conceivably impact virulence regulation.

## Results

### Identification of SNPs in 8325-4 compared to NCTC8325

To provide a complete inventory of genetic changes in 8325-4 compared with 8325, we deep sequenced 8325-4 using Illumina/Solexa technology. The 8325-4 strain used for this study was obtained by one of us (DF) from Simon J. Foster (University of Sheffield, UK) and is phenotypically Agr^+^ (hemolytic on sheep blood agar; for a comprehensive review of hemolytic phenotypes in the 8325 lineage, see 16).

Prior to extensive refinement, we had initially identified 84 SNPs in 8325-4 compared to the published genome sequence of NCTC8325. During the course of our analysis, however, Berscheid et al. reported that a large number of the SNPs that had been identified by sequence comparison between RN4220 and NCTC8325 [13] ultimately turned out to represent sequencing errors in the NCTC8325 genome [14]. Correction for these errors in our analysis reduced the initial number of *in silico* called SNPs in our 8325-4 strain from 84 to 40. In light of our concern for additional reference genome sequence errors, we subsequently re-sequenced all 40 candidate SNPs using appropriate PCR primers (Table S1) and genomic DNA derived from the sequenced NCTC8325 strain deposited in the NARSA repository as NRS77. We found that 20 of the 8325-4 candidate nucleotide variations were already present in NRS77, and thus most likely represented additional sequencing errors in the NCTC8325 reference genome rather than real differences between 8325 and 8325-4 (Table 1). Of note, sixteen of the corrected nucleotides were reported as SNPs in the published analysis of RN4220 [13]. The remaining 20 SNPs detected in 8325-4 were thus confirmed as *bona fide* genetic differences between 8325-4 and NCTC8325 (Table 2). Among these SNPs, 13 are non-synonymous substitutions or frameshift mutations in coding regions, 3 are synonymous substitutions, and 4 are located in intergenic regions (Table 2). Fifteen of the 20 SNPs (75%) were previously identified in the 8325-4 derived strain, SH1000, by the method of complete genome hybridization (CGH) [15].

**Table 1:** Identified errors in the NCTC8325 sequence.

Genome position	Locus tag (SAOUHSC number)	8325 annotation	8325 reference	8325 corrected	Impact of correction	Reported as SNP in RN4220 [13]
22181	Intergenic 00018 -00019		C	A		+
47649	Intergenic 00044 -00045		T	-		+
292840	00275	Hypothetical protein	G	A	silent	
448755	Intergenic 00446 - R0001		*	+A		
448771	Intergenic 00446 - R0001		T	A		
453801	R00011	Sa5SA	A	T		
649126	00661	Hypothetical protein	G	T	silent	+
841103	00877	Hypothetical protein	G	T	silent	+
841139	00877	Hypothetical protein	G	T	silent	+
1653482	01748	Tgt	G	A		
2244414	Intergenic 02416 - R0005		T	-		+
2244467	Intergenic 02416 - R0005		A	G		+
2244495	Intergenic 02416 - R0005		G	A		+
2318272	Intergenic 02512 -02515		G	A		+
2318274	Intergenic 02512 -02515		G	T		+
2318290	Intergenic 02512 -02515		C	A		+
2383630	02591	Hypothetical protein	G	T		+
2383660	02591	Hypothetical protein	G	T		+
2689048	02923	Hypothetical protein	G	T	V353L	+
2762204	02990	Hypothetical protein	T	C	N1514S	+

**Table 2:** Confirmed SNPs in 8325-4 compared to NCTC8325.

Genome position^a^	Locus tag (SAOUHSC number)	Annotation	8325 base	8325-4 base	Amino acid change	NRS135^b^	ISP794^c^ [46]	RN4220^c^ [13]	SH1000^c^ [15]
110019*	00105	Phosphonate ABC transporter, putative substrate-binding protein	C	A	V16F	+		+	+
392716	Intergenic 00389 -00390		G	A		+		+	+
405366	Intergenic 00401 -00402		A	G		+		+	
412760*	Intergenic 00411 -00412	Phenol-soluble modulin alpha 3	*	+T	L7fs	+		+	
412765*	Intergenic 00411 -00412	Phenol-soluble modulin alpha 3	C	G	A5P	+		+	+
653552	00666	Two-component sensor histidine kinase, GraS	C	G	silent	+		+	+
653801	Intergenic 00666 -00667		C	T		+		+	
751285*	00769	Preprotein translocase subunit, SecA	A	T	E449V	+		+	+
827849	00859	Hypothetical protein	A	T	T164S	+		+	+
939304*	00961	Competence transcription factor, ComK	C	T	E53K	+		+	+
954590*	00983	2-succinyl-6-hydroxy-2, 4-cyclohexadiene-1-carboxylic acid synthase/2-oxoglutarate decarboxylase	C	T	silent				+
1009713*	01041	Pyruvate dehydrogenase E1 component beta subunit, PdhB	G	C	G64A				+
1016979	01048	Spermidine/putrescine ABC transporter, permease protein, putative	G	A	E220K	+		+	+
1020577*	01053	Putative manganese transporter, MntH	G	T	S286STOP	+		+	+
1123048*	01172	Orotate phosphoribosyl transferase, PyrE	G	A	G42S	+		+	+
1358230*	01418	Alpha ketoglutarate dehydrogenase, SucA	C	T	D590N	+		+	+
2106539	02274	ABC transporter, ATP-binding protein, putative	A	T	L602F	+		+	+
2272936*	Intergenic 02448 -02449		A	T					
2678563*	02911	Hypothetical protein	T	C	silent	+	+	+	
2733480*	02971	Zinc metalloproteinase aureolysin	G	T	Q231K				+

a An asterisk indicates that the SNP was included in an extended SNP analysis.

b This study

c These columns indicate the results of published data

### Identification of larger deletions in 8325-4 compared to NCTC8325

In addition to the point mutations, we identified five larger deletions in 8325-4 compared to NCTC8325, of which three corresponded to the expected Φ11, Φ12 and Φ13 excisions (Table S2). One 63 bp deletion was located in the *sarS-spa* intergenic region of 8325-4, and was previously described by O’Neill in SH1000, but in contrast, not detected in 8325-4, most likely because of the use of an 8325-4 of a variant pedigree (described below) [15]. By PCR analysis, we confirmed the presence and absence, respectively, of the 63 bp deletion in our sequenced 8325-4 and in 8325 (NRS77).

Finally, we detected a 1.2 kb deletion first described in RN4220 as covering the region encoding the β subunit of excinuclease ABC [13]. Our PCR and sequence analysis showed, surprisingly, that the 1.2 kb region was absent not only in 8325-4 but also in 8325 (NRS77) most likely indicating another sequencing or assembly error in the 8325 reference genome. Curiously, a BLAST search of the missing 1.2 kb region identified no homologous sequences in other sequenced *S. aureus* strains, but revealed 100% nucleotide sequence identity to insertion sequence (IS) transposon elements from e.g. *Escherichia coli*. Inspection of the context of the 1.2 kb fragment (Figure S1) shows that it may have inserted in the middle of the gene encoding the β subunit of excinuclease ABC during the NCTC8325 sequencing project, perhaps in the course of shotgun cloning or BAC propagation [17]. RAST annotation of our *de novo* assembled 8325-4 contigs and the published RN4220 contigs [13] showed that the gene encoding the β subunit of excinuclease ABC was intact in both 8325-4 and RN4220 (Figure S1).

### Variability and duplication of SA0282 homologs

**Figure 1:**
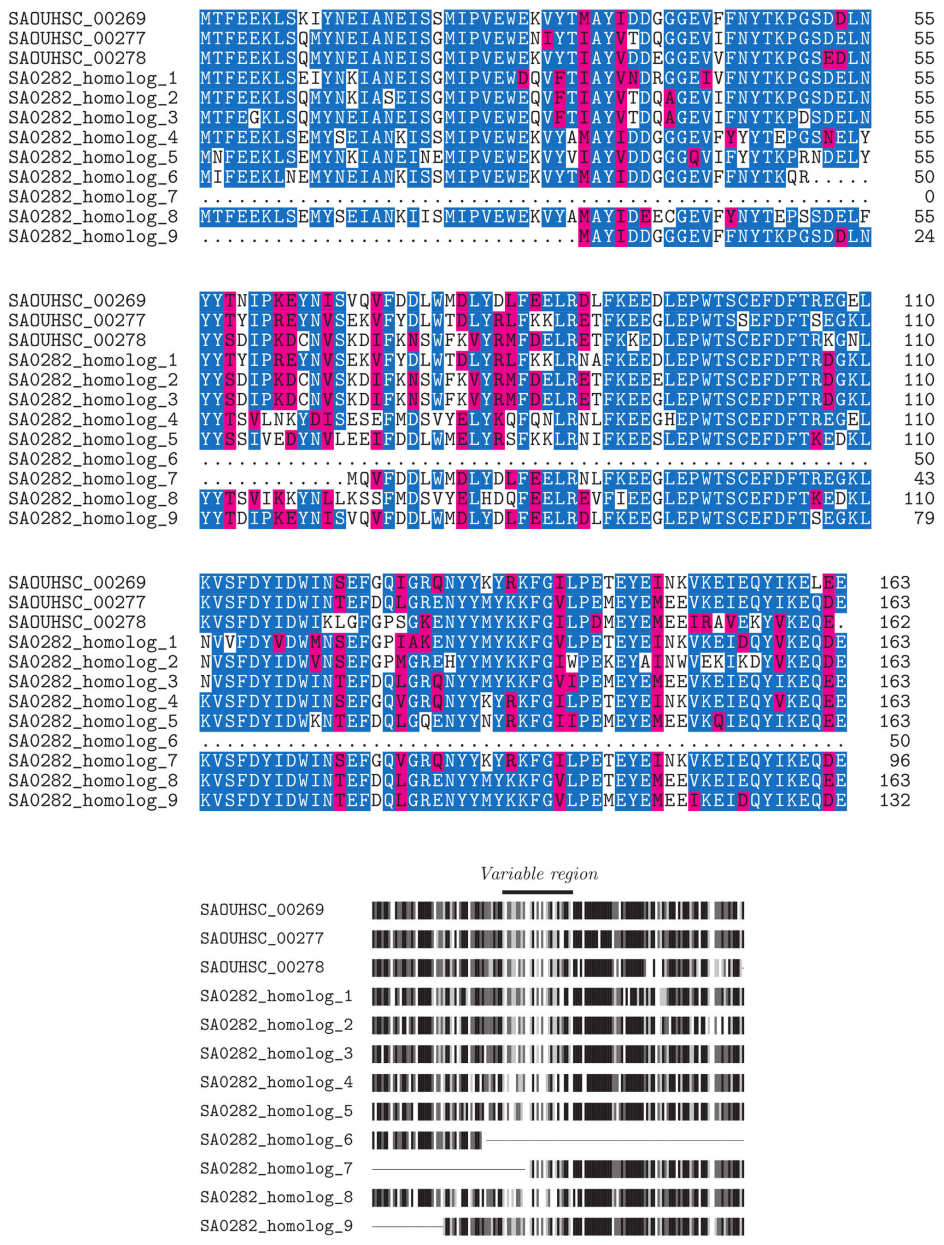
Alignment of the twelve hypothetical proteins encoded in the 8325-4 genome with homology to SA0282. The sequences were aligned using ClustalX and the alignment was visualized using the TEXshade package [51]. The top panels shows identical (blue), similar (pink) or non-conserved residues (white). The bottom panel shows each sequence on a single line with vertical gray scale lines indicating identical (black), similar (gray), or non-conserved residues (white), and with an indication of a variable region with little sequence identity.

Mapping the obtained sequencing reads to the region spanning position 292120 through 293128 in the NCTC8325 genome resulted in an anomalously high number of differences with a low quality score and an above average read coverage, collectively indicating mis-mapped reads. Three genes annotated in NCTC8325 as “hypothetical proteins” (SAOUHSC_00274, SAOUHSC_00275 and SAOUHSC_00276) are located in this region, and using the RAST server [18] they were re-annotated as “repetitive hypothetical proteins near ESAT cluster, Sa0282 homologs”, of which NCTC8325 contains six. RAST annotation of our *de novo* assembled contigs shows that 8325-4 contains nine novel SA0282 homologs in addition to three identical to SAOUHSC_00269, SAOUHSC_00277 and SAOUHSC_00278 found in 8325. None of the hypothetical proteins encoded by the total of 12 SA0282 homologs present in our sequenced 8325-4 have identical sequences, although they contain segments of highly conserved amino acid residues (Figure 1). Inspection of the whole-genome sequence of RN4220 also reveals the presence of 12 SA0282 homologs in this strain [13], however, only six of these are identical to those we describe in 8325-4, indicating the likelihood for inter-strain variability at this locus. Other sequenced *S. aureus* strains contain a variable number of SA0282 homologs, which are located immediately downstream of the Ess (ESAT-6 secretion system) gene cluster [19].

### Discovery of an 8325-4 sub-lineage

Having verified all sequence variations in our sequenced 8325-4 strain (hereafter referred to as 8325-4seq) and corrected additional sequencing and assembly errors in the 8325 reference genome, we wished to address the possibility that our sequenced strain was not representative of the majority of strains within the 8325-4 lineage. We therefore sequence verified every SNP and the 63 bp deletion in an independent 8325-4 strain (NRS135) obtained from the NARSA strain repository. We found that four of the identified SNPs and the 63 bp deletion were not present in NRS135 (Table 2), demonstrating that more genetic variability exists among 8325-4 strains than perhaps previously believed [16].

**Figure 2:**
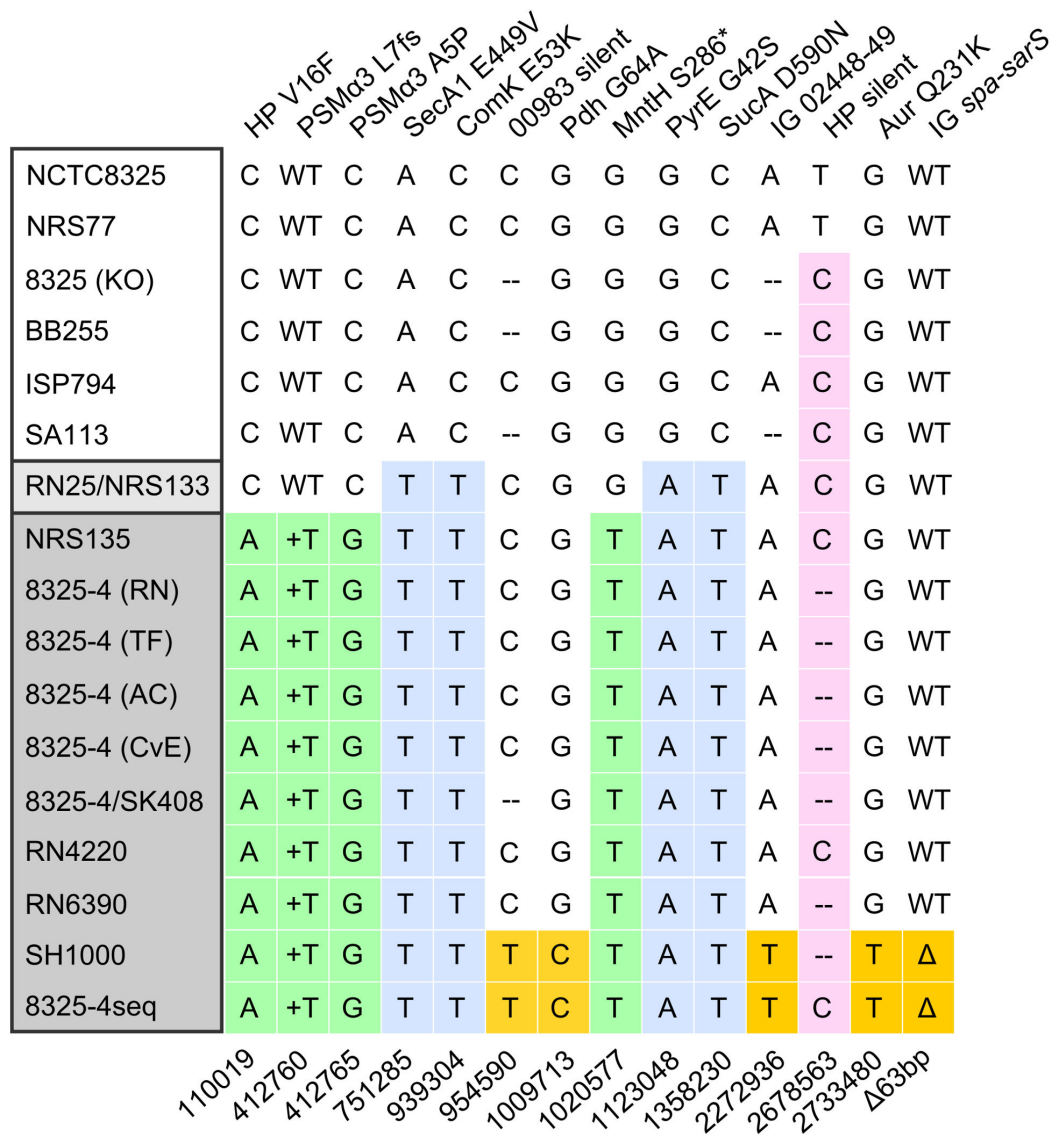
Distribution of SNPs in the *S. aureus* 8325 and 8325-4 strain lineages. Thirteen of the SNPs including the 63 bp deletion detected in 8325-4seq and described in Table 2 were analyzed by PCR from gDNA and direct sequencing for their distribution using strains collected from various laboratories worldwide. Shown in the matrix are 6 strains of 8325 pedigree group prior to prophage removal (white box), 10 strains from the 8325-4 pedigree after prophage Φ11, 12, and 13 removal (dark gray box), and one intermediate strain, RN25/NRS133, which retains prophage Φ13 (light gray box). Each SNP is indicated (top) together with coordinates from the 8325 reference genome (bottom) deposited as Genbank NC_007795.1. Strain abbreviations used are as described in Table 3. WT: wild type, +T: insertion of T, ‘ -‘ denotes SNP not tested.

In light of this finding, we next sought to determine the extent of SNP variation in a broader sampling of 8325 strains using NARSA reference strains, 8325 strains, 8325-4 strains, and the 8325-4 derived strains, RN6390, RN4220 and SH1000 (Table 3). This collection of strains has generously been provided to us from various laboratories worldwide over the course of several decades. Overall, our SNP distribution analysis included 13 of the 20 SNPs (primarily chosen to exclude silent mutations) together with the 63 bp deletion in each strain to build a SNP matrix (Figure 2).

**Table 3:** Bacterial strains used in the study.

Strains	Abbreviation	Source
PS47		M. Heck (National Institute of Public Health and the Environment, the Netherlands)
8325 (BB255)		B. Berger-Bächi (University of Zürich, Switzerland) [52]
8325	KO	K. Ohlsen (University of Würzburg, Germany)
8325 (NRS77)		NARSA (www. narsa.net)
8325 (RN1)		R. Novick (New York University, NY, USA)
SA113		S. Cramton (University of Tübingen, Germany)
ISP794 (Sequenced in [46])		P. Pattee (Iowa State University, IA, USA)
RN25 (NRS133)		NARSA (www. narsa.net)
8325-4 (RN450)	RN	R. Novick (New York University, NY, USA)
8325-4/SK408		G. Mahairas (Iowa State University, IA, USA) [53]
8325-4	TF	T. Foster (Trinity College, Ireland)
8325-4	AC	A. Cheung (Dartmouth Medical School, NH, USA)
8325-4	CvE	C. von Eiff (University of Münster, Germany)
8325-4 (Sequenced in this study)	seq	S. Foster (University of Sheffield, UK)
8325-4 (NRS135)		NARSA (www. narsa.net)
RN6390		P. McNamara (Univ. of Wisconsin-Madison, WI, USA)
RN4220		R. Novick via D. Hooper (Harvard Medical School, MA, USA)
SH1000		S. Foster (University of Sheffield, UK)
RA1		[45]
PC1070		S. Foster (University of Sheffield, UK) [20]
PC161		S. Foster (University of Sheffield, UK) [20]
PC322		S. Foster (University of Sheffield, UK) [20]
8325-4 ∆*spx*		[21]
8325-4 *spx*-c		[21]

**Figure 3:**
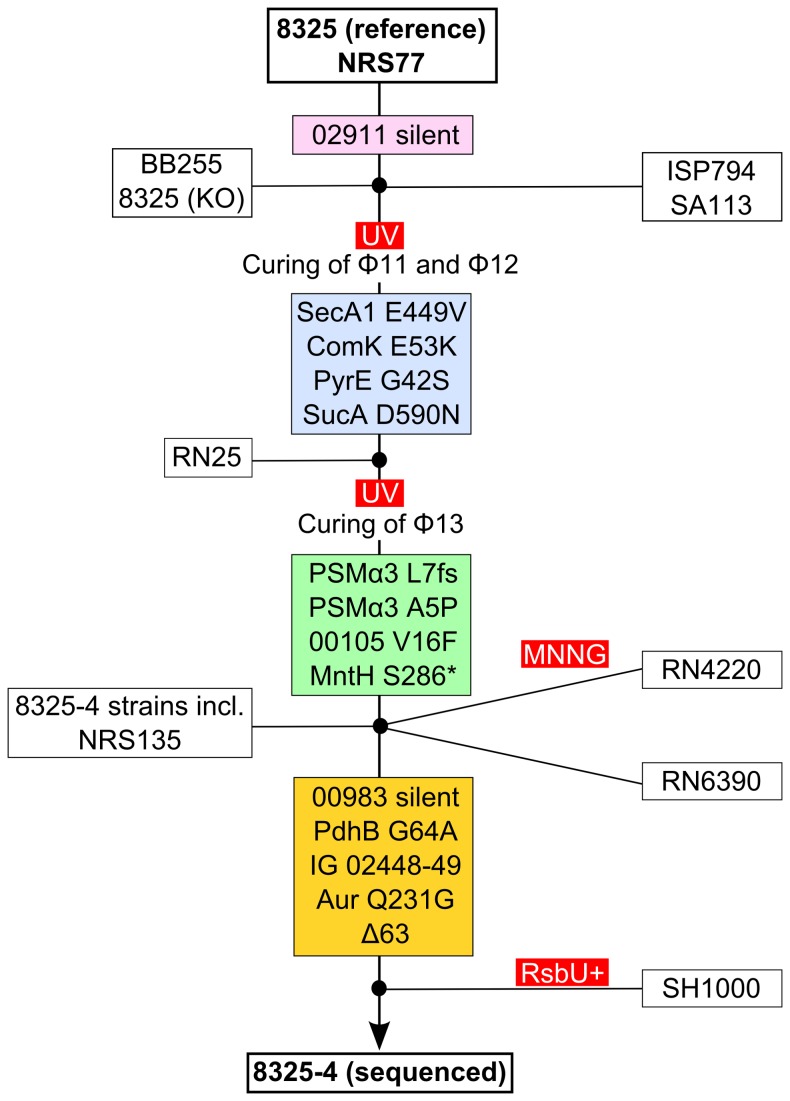
High resolution 8325 pedigree based on the SNP analysis depicted in Figure 2. Strains are shown in white boxes, mutagenesis treatments are shown in red boxes, and SNPs are shown in colored boxes, color coded as in Figure 2, on the line indicating strain evolution from 8325 to 8325-4seq. The placement of the SNPs along the line indicates their entry point into the pedigree. Five digit numbers indicate SAOUHSC locus tags. IG: intergenic.

Based on the SNP matrix, we deduced a high-resolution 8325 pedigree (Figure 3) that identifies the likely entry point for most of the SNPs that have been introduced during the course of evolution from 8325 (NRS77) to 8325-4seq. What is strikingly clear from the resolved pedigree is that two lineages of 8325-4 strains exist: a main lineage comprising several 8325-4 strains, RN4220 and RN6390, and a sub-lineage comprising 8325-4seq and SH1000. We find strains of this sub-lineage spanning at least eight years, for example PC1070, PC161 and PC322 [20] as well as Δ*spx* and *spx*-c [21], suggesting the possibility that it may be widely disseminated. Furthermore, the resolved pedigree now provides clear evidence for the suspicion that mutations had been introduced during the two successive cycles of UV irradiation that was originally applied to remove prophages. Lastly, we note that the sequenced 8325 strain differs from the main 8325 group by a silent mutation (Figures 2 and 3). It is important to emphasize that our current analysis does not exclude the presence of additional mutations in the pedigree that are not present in 8325-4seq, even for the non-mutagenized strains.

### Importance of the SNPs in the PSM α operon and the 63 bp deletion between *sarS* and spa

**Figure 4:**
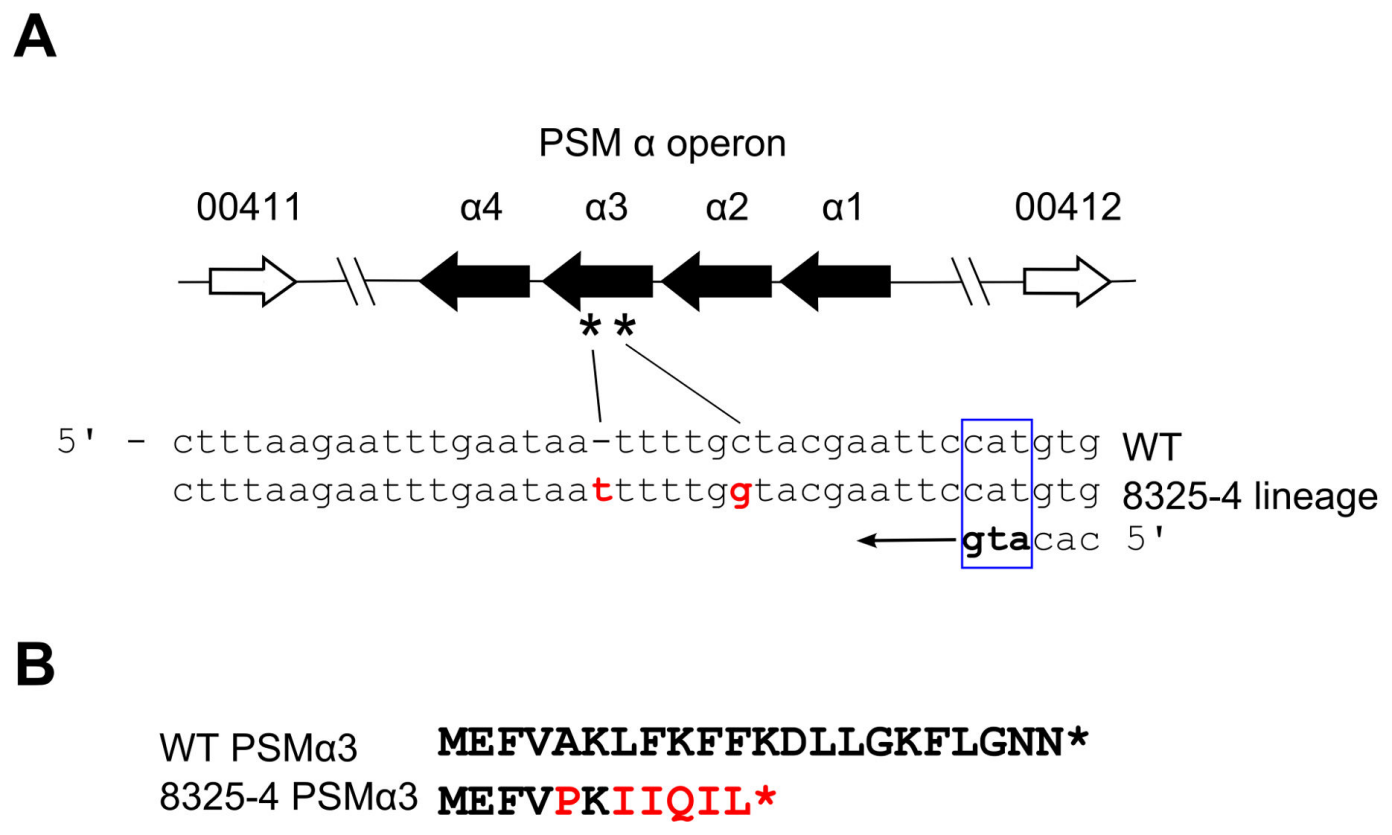
Organization of the phenol soluble modulin αoperon showing the location of mutations (A5P, and L7 frameshift) in PSMα3 detected in the 8325-4 lineage. (A) Shown is the non-annotated intergenic genomic region of *S*. *aureus* NCTC8325 sequence between sequence tags SAOUHSC_00411 and SAOUHSC_00412 encoding the PSMα operon. The position of the two detected mutations are shown in red on the antisense strand starting with NCTC8325 coordinate 412741, and the start codon is boxed in blue. (B) wild type and mutated PSMα3 translation products.

The SNPs found at positions 412760 and 412765, and the 63 bp deletion in the *spa-sarS* intergenic region merit particular attention. In NCTC8325, no genes are annotated in the 1281 bp intergenic region between ordered sequence tags SAOUHSC_00411 and SAOUHSC_00412 in which the two nearly adjacent SNPs were identified. Such large intergenic regions are unusual and thus we considered the possibility that the region had been inadequately annotated. Six-frame translation of this entire region and detailed re-examination revealed four ORFs identical to the genes in the phenol soluble modulin (PSM) α operon [22] found ubiquitously in *S. aureus* strains. Remarkably, the two identified SNPs in this region result in an amino-acid substitution (A5P), and a frameshift (L7fs) causing a premature stop codon in PSMα3 (Figure 4). PSMs have a demonstrated role in virulence of *S. aureus*, particularly in CA-MRSA strains by virtue of their potent chemotactic and pro-inflammatory traits, and their capacity to lyse neutrophils [22,23]. PSMs also display surfactant properties that permit *S. aureus* to spread on moist surfaces and modulate biofilm architecture [24,25]. *S. aureus* encodes seven core genome PSMs (the α- and β-operons, together with *hld*, encoding hemoylsin δ, embedded within the RNAIII transcript), with an eighth PSM encoded on a horizontally acquired SCCmec-element [22,23]. Notably, PSMα3 is the most potent neutrophil chemotactic and neutrophil lysing agent among the PSMs tested *in vitro* [22], and, although its effects are attenuated or inactivated by serum, it has been speculated that PSMs may nevertheless contribute to virulence of intracellular *S. aureus* [26,27].

Remarkably, our extensive SNP analysis shows that all tested 8325-4 strains, but not the immediate precursor still possessing Φ13 prophage, strain RN25, possess both SNPs within PSMα3 (Figures 2 and 3). On the basis of this evidence, it is reasonable to speculate, that all 8325-4 strains, including widely used derivatives such as RN6390 and SH1000, lack a functional PSMα3. Consequently, 8325-4 strains clearly lack a virulence factor compared with 8325. It remains unknown whether the truncated PSMα3 possess any biological function or whether the alternative translational termination exerts any polar effects within the operon.

**Figure 5:**
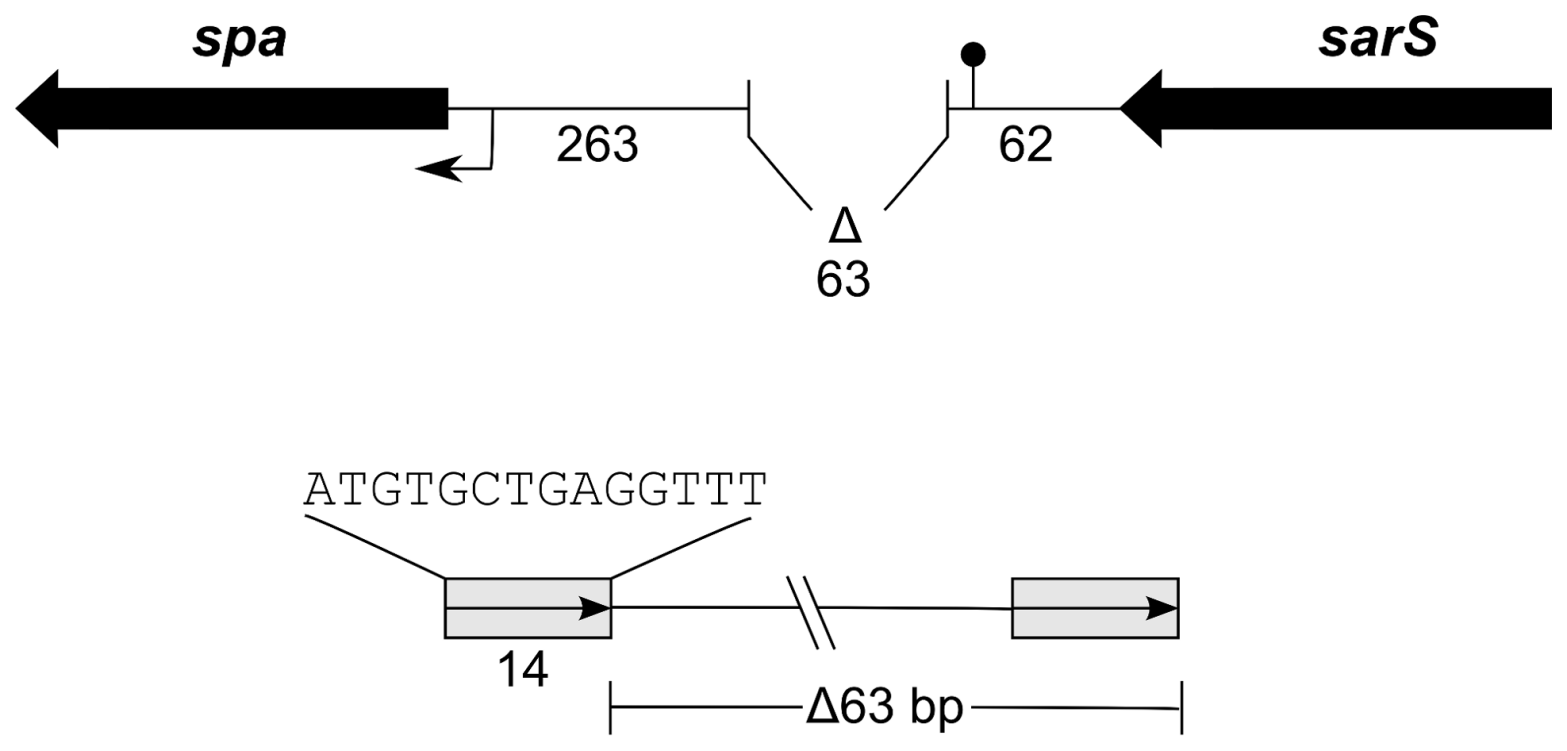
Diagram of the *spa-sarS* region showing the location of the 63 bp deletion (top panel) detected in a sub-lineage of 8325-4 strains. The *sarS* transcriptional terminator as described in [28] is shown as a filled circle. Sequence analysis shows that the deletion arose by precise excision of sequence flanked by 14 bp direct repeats (bottom panel).

The 63 bp deletion that was previously detected in SH1000 [15], and detected by us in a sub-lineage of 8325-4, is located in the divergent intergenic region between *spa*, encoding the cell-wall anchored virulence factor protein A, and *sarS*, encoding the transcriptional regulator, SarS (also called SarH1). The deletion is immediately adjacent to the *sarS* transcriptional terminator without affecting its invert repeat sequences [28]. The deletion is also apparently upstream of all known *spa* promoter elements encompassing binding sites for SarA and SarS [29]. Sequence analysis shows that the deletion most likely arose by precise recombination within the 14 bp direct repeats and excision loss (Figure 5). Apart from those strains mentioned above (e.g. PC1070, Δ*spx*), we did not detect the 63 bp deletion in any other sequenced staphylococcal strains in the nucleotide database using BLAST analysis. What conditions might govern the excision process are unknown, but nevertheless points to the potential of genome instability in this region. Repeat regions within *spa* are known to be of variable length and thus recombination within this immediate region could conceivably contribute to this instability.

**Figure 6:**
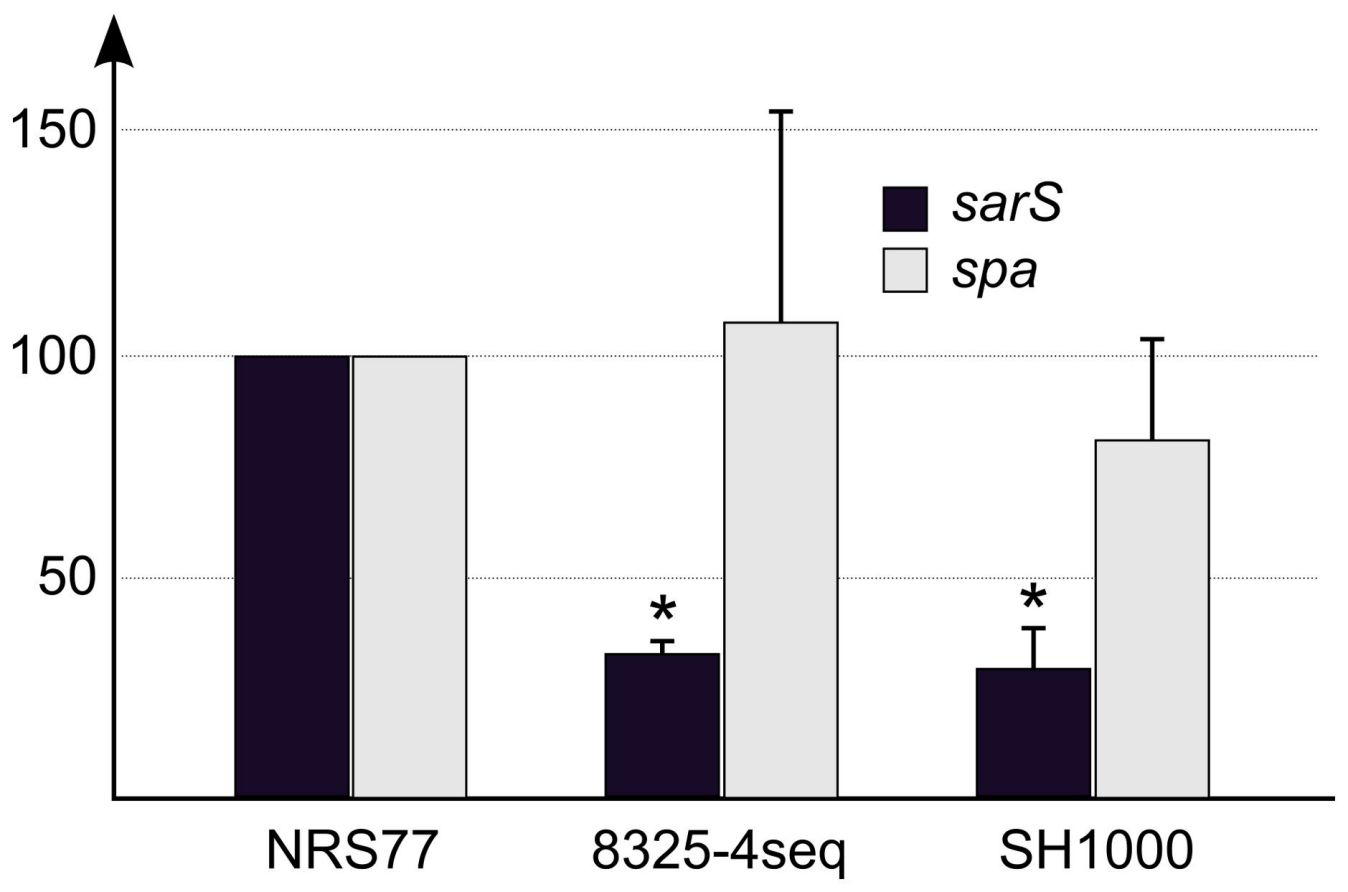
Assessment of 63 bp deletion in the *spa-sarS* intergenic region on the flanking genes by qRT-PCR analysis. RNA extracts were prepared following entry to stationary phase (Materials and Methods). RNA was obtained from the various strains indicated and experiments normalized to 16S RNA followed by comparison to NRS77 (8325) fixed as reference control. * Indicates significant difference determined by Student’s two tail *t* test using three independent sample determinations. Pairwise comparisons revealed a pronounced reduction (75%) in *sarS* mRNA in strains 8325-4 (sequenced in this study) and SH1000, both harboring the deletion, compared with NRS77 with wild type intergenic sequence (*P*< 0.0001, respectively). No significant changes in *spa* mRNA were noted for 8325-4 or SH1000. No significant changes were observed with RNA obtained during exponential phase growth of the same cultures. All strains used for analysis gave clear zones of hemolysis on sheep blood agar and were *agrA* sequence 7A [16].

The discovery in SH1000 of the 63 bp deletion led to the proposal that it may alter expression of *spa*, although this possibility was not experimentally addressed [15]. Although the deletion maps outside of the known regulatory elements [28,29], the loss of six helical DNA turns could conceivably interfere with the regulation of gene expression of either *spa* or *sarS*. To address this question, we compared the steady-state levels in stationary phase of either *spa* or *sarS* mRNAs in two strains containing the deletion (8325-4seq and SH1000) with the levels in 8325 (NRS77), which does not contain the deletion. Importantly, all three strains display Agr^+^ phenotypes on sheep blood agar, and are AgrA-7A [16], indicating that results on this particular aspect of gene regulation are comparable in the three strains. Pairwise comparisons revealed a pronounced significant reduction (75%) in *sarS* mRNA in strains 8325-4seq and SH1000, both harboring the deletion, compared with NRS77 with the wild-type intergenic sequence (Figure 6). Therefore, it is likely that the 63 bp deletion affects gene expression of *sarS*, and thus may have an additional impact on central aspects of virulence regulation in the sub-lineage of the 8325-4 strains. In contrast with these findings, we found no significant changes in *spa* or *sarS* steady-state transcripts using RNA prepared during exponential phase growth prior to the onset of the Agr-mediated quorum sensing circuitry (data not shown). High variance was noted in multiple independent sample preparations precluding any meaningful analysis.

### Phenotypic variation in the 8325-lineage and agr instability

**Figure 7:**
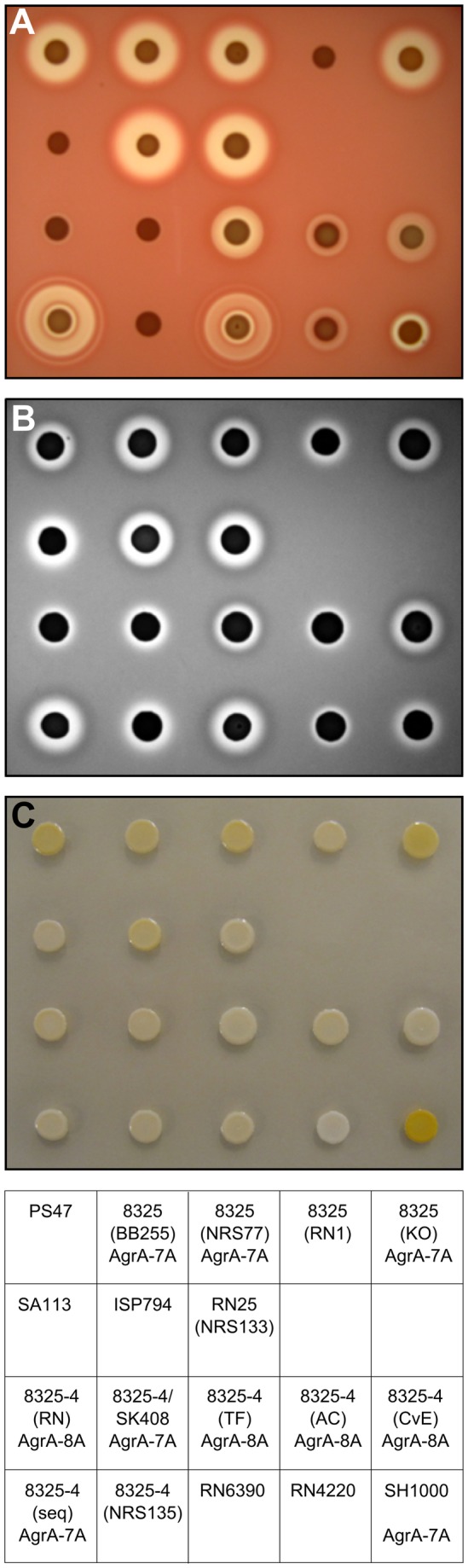
Phenotypic characteristics of strains used in this study. Panel **A**: hemolysis on sheep blood agar; panel **B**: secreted proteolytic activity on casein agar; panel **C**: pigmentation. The bottom panel indicates the strain (Table 3) together with the AgrA sequence type [16] where noted.

The disruption of the PSMα3 gene and the reduced *sarS* expression suggest variability in virulence expression among 8325-derived strains, in addition to the previously described disregulation of virulence factors among 8325-4 strains caused by AgrA instability [16]. Furthermore, we observed heterogeneity in our assembled strain set with regard to hemolysis, protease activity, and pigmentation (Figure 7) indicating a high likelihood of additional genetic changes that has arisen during the course of propagation and dissemination of these strains. We confirmed the *agrA* slippery T sequence in a majority of our 8325-4 strains (Figure 7), but we also uncovered evidence for instability in 8325 strains in contrast to what was noted previously [16]. Three of our main stocks of 8325 strains, which were obtained independently from two different laboratories over a span of a decade, contained a mixture of hemolytic and non-hemolytic colonies when plated on sheep blood agar (Figure S2). We did not detect AgrA slippery sequence in 8325 (KO) indicating that other sources of *agr* instability were likely operating to give the hemolytic negative phenotype. In most cases, we did not investigate the molecular cause further. We also noted instability in the ISP794 derivative, RA1, which could, however, be traced to a complete spontaneous deletion of the region encompassing RNAIII (data not shown). *agr* instability has been noted upon sustained cultivation of laboratory strains [30] and expression of RNAIII has been shown to impose a significant fitness cost on *S. aureus* Newman cells, which may explain the frequent isolation of mutants defective at this locus [31]. The variability within the 8325 strain lineage described in this study thus suggests multiple potential routes to disregulation of virulence factors in the entire pedigree of 8325 and 8325-4.

### Bioinformatics analyses to predict possible consequences of remaining SNPs

To predict possible consequences of the remaining genetic changes in 8325-4, and as a first step to evaluate their potential impact on virulence, regulation and physiology, we examined the context of each SNP using proteomic database mining tools such as eggNOG, Pfam, UNIprot, BLAST and RAST.

Several of the SNPs in coding regions can be predicted to have a potential impact on protein function. MntH (SAOUHSC_01053) is a putative Mn(II) transporter, and is predicted to be a 450 amino acid transmembrane protein with multiple membrane spanning regions. Therefore, the stop codon at position 286 most likely completely disrupts the protein’s tertiary structure and function. Mn(II) mostly assists in the defense against oxygen radicals and its transport could thus be considered a clear survival advantage. Our data in Figures 2 and 3, suggests that disruption of *mntH* is a feature of all 8325-4 strains and thus predicts a potential impact on Mn(II) uptake, and by extension, on pathogenesis in this strain lineage. Notably, *S. aureus* encodes two Mn(II) transport systems, *mntABC* and *mntH*, under the control of MntR [32]. However, genetic dissection of this system was performed with 8325-4 derived strains leaving open the possibility that the phenotype of the engineered *mntH* disruption has not yet been clearly defined.

Alignment of Sec A1 (SAOUHSC_00769) homologs shows that all members of this protein family have an acidic amino acid (aspartate or glutamate) at the mutated position (VETSEYISN), thus a substitution to valine might alter the proper function of Sec A1. The SecAEY secretion apparatus is essential so such non-synonomous substitution might necessarily have little or a subtle effect. On the basis of structural studies with the *E. coli* SecA preprotein translocase, the equivalent site of mutation is amino acid 463 found at the midpoint of α-helix 17 and comprising part of IRA2, the intermolecular regulator of ATPase subdomain [33].

Locus-tag SAOUHSC_00961 harbors an E53K mutation, and is annotated in NCTC8325 as ‘hypothetical protein’, but re-annotation shows that this gene encodes the presumptive *S. aureus* ortholog of the competence regulating transcription factor, ComK, of *B. subtilis* [34], also called competence transcription factor. Alignments show that amino acids found at the mutated position are either negatively charged (aspartate or glutamate) or neutral (alanine or glycine), but there is no evidence of charge reversal substitutions (glutamate to lysine). In *B. subtilis*, ComK is thought to act as a dimer, and following binding to specific DNA sequences (K-boxes) and formation of a tetramer, its mode of transcriptional activation is by bending DNA and facilitating RNAP binding [35,36]. Given the need for DNA binding, dimer interface, tetamerization, and perhaps RNAP interaction, a charge reversal mutation would be expected to have a functional consequence. *S. aureus* has not been traditionally known as competent, but a recent study now demonstrates that naturally competent *S. aureus* cells can be obtained by activation of a cryptic sigma factor, SigH [37]. Whether and to what extent ComK contributes to competence in *S. aureus* or performs other regulatory roles requires additional study.

The putative spermidine/putrescine transporter (SAOUHSC_01048) has a glutamate at the mutated position that is highly conserved in staphylococci. There is no evidence for charge reversal and thus the E220K substitution would be predicted to have a functional consequence. Similarly, SAOUHSC_01418, *sucA*, encodes alpha ketoglutarate dehydrogenase and alignments of SucA homologs shows clear evidence for only an acidic residue (glutamate or aspartate) at the mutated position in Firmicutes. Thus, D590N would be predicted to have a consequence for SucA function.

Analysis of the context of the four intergenic SNPs shows they are located in or close to demonstrated or putative regulatory elements and thus can be predicted to have a potential impact on gene expression. Notably, the SNP at position 653801 is located in the 144 bp intergenic region between *graS* and *vraF*, 138 bp upstream of ATG of VraF and 6 bp downstream from the GraS stop codon. GraR, the response regulator of the GraSR two-component system, regulates transcription of the *vraFG* operon via a conserved binding site located in the extreme C-terminal coding region of GraS [38]. The SNP is one helical turn downstream from this GraR binding site, and thus clearly within the boundaries of the *vraFG* promoter regulatory elements. The SNP at position 392716 is in a 364 bp intergenic region 255 bp upstream of the ATG of SAOUHSC_00390, a putative exotoxin.

The SNP at position 405366 is in a 338 bp intergenic region between divergently transcribed genes, encoding a hypothetical protein (SAOUHSC_00401), and a putative uncharacterized lipoprotein (SAOUHSC_00402), respectively. The last intergenic SNP at position 2272936 is located in a 251 bp intergenic region 32 bp upstream of the putative start codon of SAOUHSC_02448, and thus within likely promoter elements. SAOUHSC_02448 encodes a putative cell surface hydrolase.

Seven SNPs in coding regions are predicted to be of less importance either because they are silent, because the amino acid at the SNP position is not well conserved within the Firmicutes family (V16 in SAOUHSC_00105, G64 in PdhB, Q231 in Aur), or because the amino acid substitution resulting from the SNP was conserved at that position (G42S in PyrE). The SNP positions in SAOUHSC_00859 and SAOUHSC_02274 are located in regions that are not highly conserved; however the observed substitutions are not seen in Firmicutes, so we cannot confidently predict whether these SNPs would impact protein function.

## Discussion

In this study, we have deep sequenced *S. aureus* strain 8325-4 in an effort to understand to what extent mutations arose during the course of its preparation and subsequent use worldwide. The progression from 8325 to 8325-4 required two successive exposures to ultraviolet light for prophage removal, and many laboratories have assumed that 8325-4 is a wild type strain because of its close relation to 8325. Derivative strains, such as the restriction-defective RN4220, are documented to have been prepared by chemical mutagenesis, and accordingly, analysis of its genome sequence has revealed a broad range of mutations [13,14].

We have discovered numerous mutations that define at least two distinct pedigrees of *S. aureus* 8325-4 and clearly distinguish it from its immediate precursor 8325. Notable among our reported findings, is the discovery that all tested strains derived from and including 8325-4 lack PSMα3 and possess additional non-synonomous mutations with a potential impact on virulence. A sub-lineage of 8325-4, detected in a small collection of published strains, which include SH1000, contains a 63 bp deletion in the *spa-sarS* intergenic region that results in significantly reduced *sarS* steady state transcripts in post-exponential phase compared with 8325, suggesting a further potential for virulence factor disregulation. Overall, our present study integrates data obtained from detailed published studies of 8325, RN4220 and SH1000 [10,13-15,17] and helps to further refine our understanding and assessment of numerous strains widely used in *S. aureus* research.

It is important to emphasize that we have used a broad sampling of 8325-4 and its derived strains to determine the extent of SNP radiation out of concern that our particular sequenced strain was not truly representative of the mutation spectrum in this lineage. Our findings show that the mutations are indeed widespread and could be used with confidence to construct a refined pedigree of the 8325 lineage. Furthermore, one of our discoveries is that 16 mutations, previously described in RN4220, are in fact characteristic of 8325-4 strains and arose prior to MMNG mutagenesis and the lineage divergence of RN4220.

Among the 20 total SNPs discovered in our sequenced 8325-4 strain, nine are transitions, 10 are transversions, and one is a nucleotide insertion. Ultraviolet light-induced mutagenesis could account for all of these mutation types [39], although we cannot of course rule out that some were the consequence of spontaneous events. It is curious that we detected two mutations mapping within the extremely short PSMα3 reading frame (encoding 22 amino acids). The dual mutation is not present in RN25 (8325 harboring the Φ13 lysogen), the immediate precursor of all members of the 8325-4 lineage, suggesting that the mutations most likely arose simultaneously, or near-simultaneously following the second round of UV exposure to remove Φ13. Given the broad conservation of genes encoding staphylococcal PSMs and their diverse roles described to date in biofilm structure, colony spreading, and virulence [24,25], it is clearly a priority to determine the collective and individual functions of these remarkable small peptides.

We presented evidence that 8325-4 strains harboring the 63 bp *spa-sarS* intergenic region deletion display reduced *sarS* transcript levels in post exponential phase compared with 8325. The role of SarS (also called SarH1) as a regulator of virulence factors is incompletely understood and there is evidence for interstrain differences in *sarS* transcript levels detected in clinical strains distinct from the 8325 lineage [40]. SarS is thought to act both positively as an activator of *spa*, encoding protein A, and negatively as a repressor of *hla* encoding hemolysin α [28,41]. Current models show SarS to be regulated by a complex control circuitry including *agr*, the global accessory regulator SarA, SarT, and possibly the ClpXP proteolytic/chaperone complex [42,43], for review, see 44. The full extent of genes, especially exoproteins and surface virulence factors, under the control of SarS is unknown. Strains of the 8325 lineage lack a functional TcaR and consequently already display reduced levels of *sarS* [12]. Clearly, further reducing *sarS* levels in some 8325-4 strains would predictably have multiple consequences. Deciphering these effects arising from the modulation of SarS levels will be an important objective in future studies.

Apart from the absence of PSMα3, the premature stop codon in *mntH* encoding one of one of two Mn(II) transporters that could affect oxygen radical scavenging [32], and the 8325-4 sub-lineage 63 bp deletion and its effect on *sarS* transcription, the mutations we describe have no proven impact or link to virulence or protein function to our knowledge. A number of charge reversal mutations were noted in our analysis and assessment of the impact of these and other changes await future experimental study.

The twelve near-identical SA0282 homologs (68-70% amino acid identity, approximately 85% similar overall) encoded downstream of the *ess* cluster have been suggested to be possible substrates of the Ess secretion system [19]. The function of the SA0282 homologs annotated with a domain of unknown function (DUF600) is unknown. There must exist some selection pressure for the retention of these putatively secreted glutamic acid-rich small basic (pI <4) proteins displaying both multiple copies and variability.

Our sequenced 8325-4 strain is very likely highly related to the immediate 8325-4 precursor that was used to repair *rsbU* and create SH1000 [10]. A number of the mutations that we describe in our present work were detected by complete genome hybridization analysis (CGH) of SH1000, together with comparison with a reference 8325-4 strain likely derived from the alternative 8325-4 pedigree [15]. Our deep sequencing of the SH1000 close precursor thus permits us to now estimate that CGH is a robust method for SNP discovery, capable of detecting 75% of the sequence-verified mutations. Limitations of the CGH method have been noted however, notably the absence of detection of the *rsbU* 11 bp deletion [15]. Advances in deep sequencing technology and decreasing costs may strongly favor large-scale future SNP discovery by sequencing methodology.

RN4220 is often used as an intermediate plasmid host during genetic manipulation, and both RN4220 and 8325-4 have been convenient hosts for numerous gene disruptions. In recent years, a large number of defined disruptions have been transferred to other genetic backgrounds by generalized bacterial transduction. However, backcrossing engineered mutations made in RN4220 or 8325-4 to unrelated *S. aureus* strains could conceivably result in co-transfer of linked SNPs to the new hosts. The availability now of detailed whole genome sequences of both RN4220 and 8325-4 thus represents an invaluable reference resource when designing and performing carefully controlled genetic studies in *S. aureus*.

At least three discoveries have previously cast some doubt on the suitability of 8325-4 as a wild type strain: *rsbU* disruption, *tcaR* truncation, and *agr* instability; in many cases the latter arising from an *agrA* slippery T sequence [16]. Our study now adds additional evidence that 8325-4 should not be considered wild type. Repair of TcaR and RsbU defects that also occur in 8325 have given rise to 8325-derived strains named HG001-3, which are promoted as excellent candidates for model strains [5]. A wide range of alternative strains have also been used or proposed in published studies (for example, Newman, UAMS-1, COL, USA300), but surprisingly most are defective in at least one regulatory or global sensory pathway.

An *agr* instability (measured by simple hemolytic assay on blood agar plates) has been reported to be rare in 8325 as opposed to 8325-4 [16]. An *agr* instability in clinical isolates has been noted as a consequence of in vitro serial passage and may be widespread, however [30]. One additional discovery in our work was the detection of *agr* instability in two independent 8325 stocks for which we could isolate single colonies of hemolytic positive and negative phenotypes. Previous unpublished work in our laboratory using the 8325-derived ISP794 strain RAI also showed similar *agr* instability [45]. Collectively, these observations led us to consider that *agr* instability may be an issue with 8325 strains, and reiterates the call for caution during laboratory cultivation of all *S. aureus* strains used for genetic and metabolic analysis.

## Materials and Methods

### Strains

The strains used in this study are derivatives of *S. aureus* 8325 obtained from laboratories worldwide (see Table 3). NRS77, NRS133, and NRS135 were obtained through the network of Antimicrobial Resistance in *Staphylococcus aureus* (NARSA) program supported under NIAID/NIH contract # HHSN272200700055C.

### Genome sequencing and SNP analysis

Genomic DNA was prepared from an overnight culture of 8325-4 grown in MHB at 37°C, as described previously [46], and sequenced on an Illumina Hi-Seq 2000 instrument (Illumina; Fasteris, SA, Geneva, Switzerland). Paired-end reads (59.2 million 100-bp) were mapped on the NCTC8325 genome sequence (NCBI accession NC_007795 [17]) using Burrows-Wheeler Alignment Tool [47] giving a raw coverage depth of approx. 2100-fold. Mapping covered 95.3% of NCTC8325 (99.95% when corrected for prophage loss). Variant base calling relative to NCTC8325 was performed with SAMTOOLS and the CLC genomics workbench. SNPs supported by less than 99% of the reads, and all single nucleotide deletions and insertions (indels) were re-examined by capillary sequencing. The obtained reads were also *de novo* assembled using Velvet [48] into 114 contigs (sum = 2.67 Mb, N_50_ = 95.9 kb, max = 194 kb; see Sequence S1). The 8325-4 contigs and the NCTC8325 sequence were annotated or re-annotated, respectively, using the RAST server [18].

### Total RNA extraction

Overnight bacterial cultures were diluted in MHB (1/100) and grown at 37°C with agitation to exponential or post-exponential phases as determined by pilot growth curves. Bacteria were harvested and RNA extraction was performed as previously described [49]. The absence of contaminating DNA was always verified for every experiment by PCR using qRT-PCR probes in the absence of reverse transcription.

### Quantitative real-time qRT-PCR

The mRNA levels were determined by quantitative RT-PCR (qRT-PCR) using the one-step reverse transcriptase qPCR Master Mix Kit (Eurogentec, Seraing, Belgium) as described [50]. Primers and probes were designed using PrimerExpress software (version 1.5; Applied Biosystems) and obtained from Eurogentec (see Table S3). Reverse transcription and PCR were performed using primers and probes at a concentration of 0.2 and 0.1 μM, respectively. All mRNA levels were normalized on the basis of their 16S rRNA levels, which were assayed in each round of qRT-PCR as internal controls as described [50].

The statistical significance of strain-specific differences in normalized cycle threshold (*C*_*T*_) values of each transcript was evaluated by Student’s paired *t* test, and data were considered significant when *P* was <0.05.

### SNP and AgrA verification

Genomic DNA was prepared from strains listed in Table 1 as above and used as a template for polymerase chain reaction (PCR) amplification of the region surrounding each suspected nucleotide change, or *agrA* reading frame. Upstream and downstream primers for SNP verification (Table S1) were designed to create convenient fragment sizes (100-600 bp) for subsequent agarose gel purification and direct Sanger sequence analysis using either PCR driver primer.

### Assays for hemolysis, proteolysis and pigmentation

Extracellular proteolytic activity was assayed by spotting 5 μl of an overnight culture grown in TSB at 37°C on plates containing 10% skimmed milk and incubating for 24 h at 37°C. Pigmentation was assayed by letting these plates incubate at room temperature for one more day. Hemolysis was assayed by spotting 5 μl of an overnight culture grown in TSB at 37°C on plates containing 5% sheep blood agar and incubating for 24 h at 37°C followed by 24h at room temperature.

## Supporting Information

Figure S1 **Organization of the genes encoding the α and β subunits of excinuclease ABC, and the 1.2 kb inserted fragment.** The top panel shows the region in the 8325 reference genome, and the bottom panel shows the region in 8325-4, RN4220 and NRS77. Numbers indicate SAOUHSC locus-tags.
(TIF)

Figure S2 **Zones of hemolysis on sheep blood agar for various strains of the 8325 lineage.** Frozen stocks, including two independent isolates of RN1 (marked HI2901 and 2834) of the indicated strains were found to harbor mixed hemolytic and non-hemolytic variants. RAI was derived from ISP794 [45]. Despite storage at -80C, strain instability (measured by hemolytic activity) not commonly seen in NCTC8325 [16] was noted in our study in four independent strain examples.
(TIF)

Table S1 **Oligonucleotide PCR primers used for SNP analysis and indel analysis.**
(PDF)

Table S2 **Deletions in 8325-4seq compared to NCTC8325.**
(PDF)

Table S3 **Oligonucleotide q-RT-PCR primers.**
(PDF)

Sequence S1 **Sequence file 8325-4 fasta format.**
(FASTA)
