# Supplementary figures and images for: Genetic Variation in the Staphylococcus aureus 8325 Strain Lineage Revealed by Whole-Genome Sequencing
Source: PLoS One. 2013 Sep 30;8(9):e77122. doi: 10.1371/journal.pone.0077122 (PMC3786944; doi:10.1371/journal.pone.0077122)

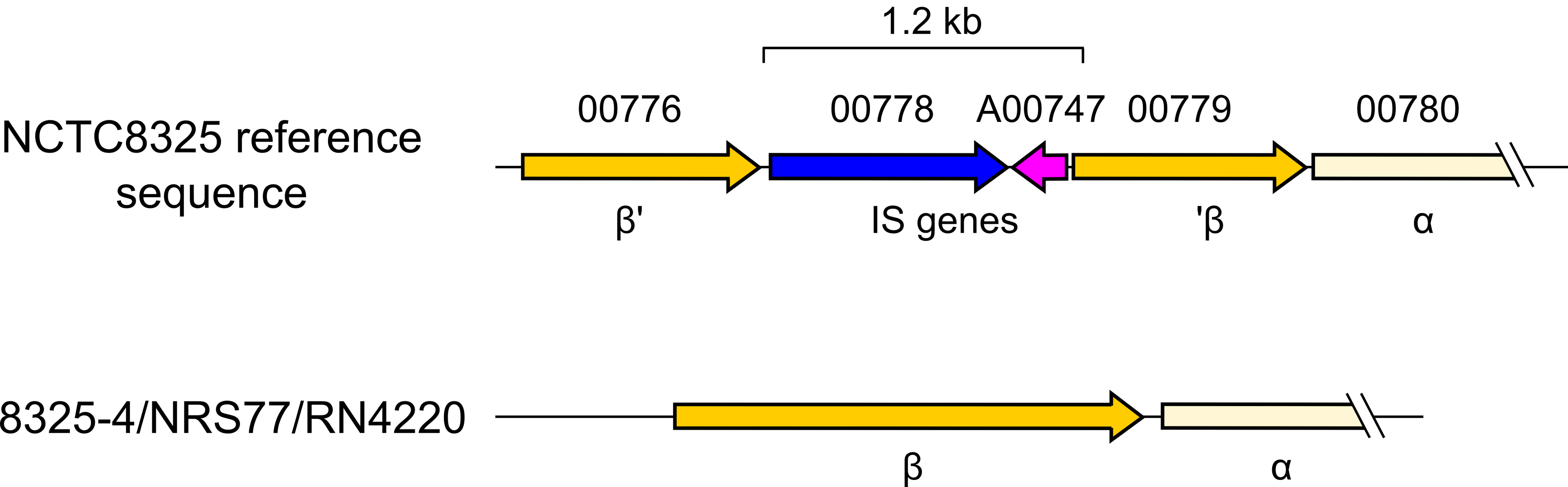

Supplement: Figure S1 — Organization of the genes encoding the α and β subunits of excinuclease ABC, and the 1.2 kb inserted fragment. The top panel shows the region in the 8325 reference genome, and the bottom panel shows the region in 8325-4, RN4220 and NRS77. Numbers indicate SAOUHSC locus-tags. (TIF) [file pone.0077122.s001.tif]

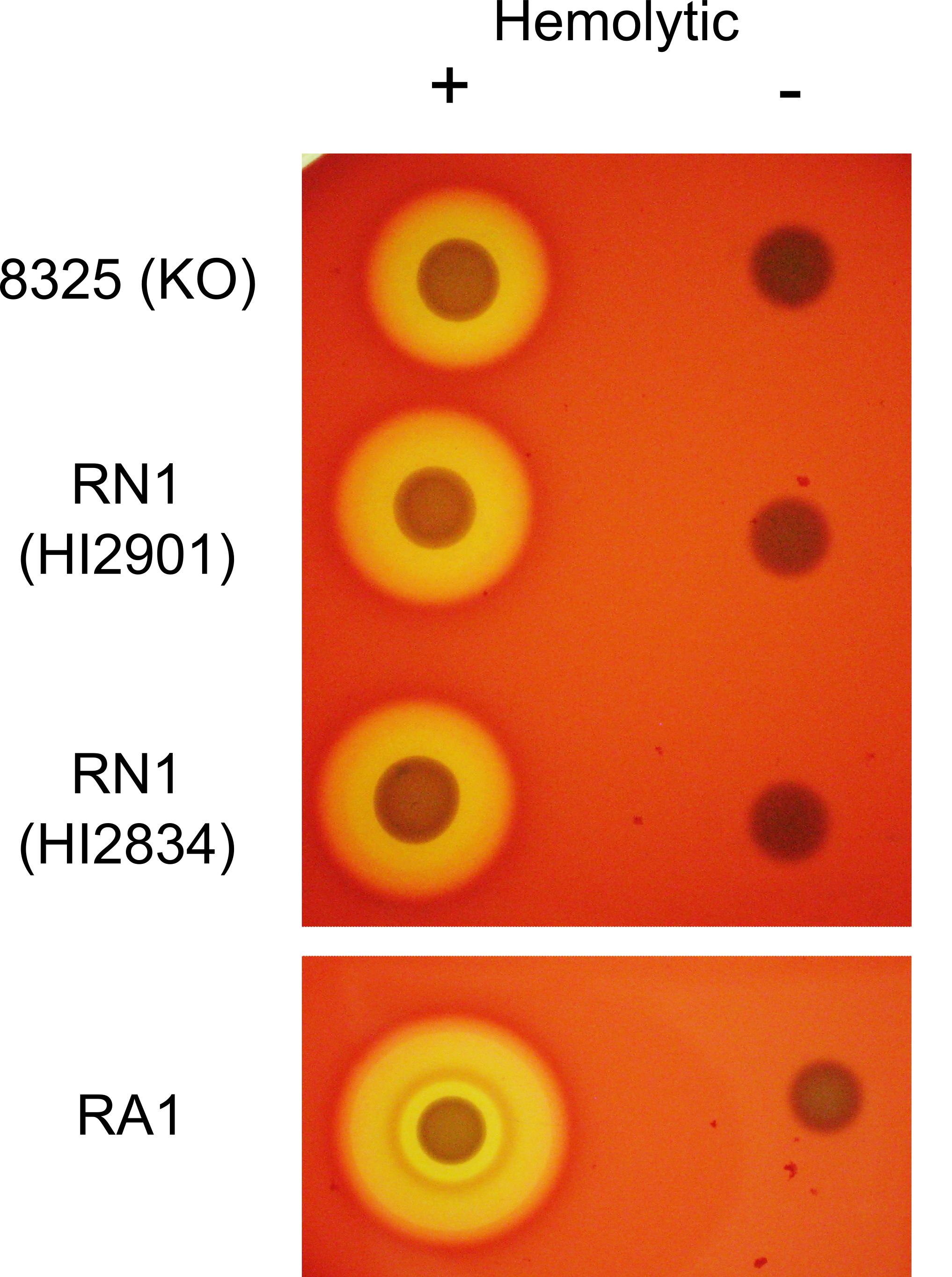

Supplement: Figure S2 — Zones of hemolysis on sheep blood agar for various strains of the 8325 lineage. Frozen stocks, including two independent isolates of RN1 (marked HI2901 and 2834) of the indicated strains were found to harbor mixed hemolytic and non-hemolytic variants. RAI was derived from ISP794 [45]. Despite storage at -80C, strain instability (measured by hemolytic activity) not commonly seen in NCTC8325 [16] was noted in our study in four independent strain examples. (TIF) [file pone.0077122.s002.tif]
