# Supplementary material for: Genetic Variation in the Staphylococcus aureus 8325 Strain Lineage Revealed by Whole-Genome Sequencing
Source: PLoS One. 2013 Sep 30;8(9):e77122. doi: 10.1371/journal.pone.0077122 (PMC3786944; doi:10.1371/journal.pone.0077122)
Supplement: Table S1 — Oligonucleotide PCR primers used for SNP analysis and indel analysis. (PDF) [file pone.0077122.s003.pdf]

# Table S1. Oligonucleotide PCR primers used for SNP and indel analysis

| Primer | Sequence 5'-3' | Primer | Sequence 5'-3' |
|---|---|---|---|
| 22181F | TGATTACTATAACCGTGGCGGCG | 292840F | GATTATATAGATTGGATAAATACAG |
| 22181R | TCCTTTTCCTTCGTCTCCCCATTG | 292840R | CATGTTATCTCCCCTATATTTCAGC |
| 2689048F | TATGTTCCATTACTCACAATACGC | 392716F | ATCACAAAGAGTAATAGTAAAAAAG |
| 2689048R | AAATGTAATTGCTGGTAACACAGG | 392716R | GTTTGATGCAATGACGTTATTGTTCC |
| 2762204F | CACTTGTACTTTCTGATGTTGAGCC | 405366F | CTCCTCTAAAAATTGTAACTCCATC |
| 2762204R | GAGAATCCACATCGACATCAACGTC | 405366R | TCCACCTTGTTTATTACTTTTGGCG |
| 110019F | GTTGATACAGACACTTTAACTGG | 412760/65F | ATAAATCAACACAAAGCAAAGCCAC |
| 110019R | GGATAAAAATTCGTTGCATATGAGC | 412760/65R | GTACAGTTAGGCAGTATTTCCCGTC |
| 751285F | AGAATGTACAATAAACTTGCGGG | 448755/71F | GATAAGGGCGTTCAGTACAGATGAC |
| 751285R | CAGAACGACCACGTAACTGGTCATC | 448755/71R | CGATCACCCTCTCAGGTCGGCTATG |
| 1020577F | AAAATGATTAAGCATACGATAACAGG | 453801F | AGCTATGTGTGGACGGGATAAGTGC |
| 1020577R | AAATCATTACAAATAACGGCATTCTC | 453801R | TCTACTCTAGCGGAACGTAAGTTGG |
| 2106539F | TTACATTGAAAAAGTTGAGGAAGCGG | 649126F | ACACCATTAATTCAAATGAATCAAGC |
| 2106539R | CTGTGGATAGCTATAATATCAGCG | 649126R | GGATTTAACGGTAATTCATTACTCGG |
| 1009713F | AGAGAAGGAGTCGAAGTAAGCC | 653552/3801F | GGTATTAGTAAAAAAGATATGCCGC |
| 1009713R | GGAATAACAACCTTTAGACCTGGAG | 653552/3801R | ATAATAGCAATAAACTCGCCTTCTTC |
| 1123048F | ACGACACCGGAAGAAGCAAGACAGC | 653552/3801F2 | TTACGTGTCACTTAAACGCATGGTC |
| 1123048R | GCTGTGACTGAAGATCCCCCTGTCG | 827849F | GGTCTATCGTAGGCATTGGAGGCGG |
| 2733480F | GACCACCGTAGTTATTAACATGCG | 827849R | TCACTTTGACACCGATTTTCGCACC |
| 2733480R | GACATTAAGTAAAGATGACGCAGCCGA | 841103/39F | ACATTGTGGTATATGTTCAGCCGGG |
| Indel75276F | AGGAAGTAAAAGGCTTAAAACCGC | 841103/39R | TGATTTCGATTAAAAATGTATCCGC |
| Indel75276R | TTTTAATTCATATGGATGACGCGCAG | AgrF2 | CAGTTAAGTATTTATTTCCTACAG |
| 47652F | AAGTAATTCAATATAAAAACAGGTG | AgrR2 | CGCCATAGGATTGTAGAGTG |
| 47652R | ATAATGTTATTTCTTCTTTTGGATGG | AgrA F1 | TTTAGAAACTGCACATACACG |
| 1653482F | CCGAACAATGCTTGATCTTCAG | 2244414/67/95F | CGAAGTTATCCCAGTCTTATAGGT |
| 1653482R | ATATGCCTGCAGTAACATACGA | 2244414/67/95R | GTTCTTGTGTTAAGCAATAAACG |
| 2318272/74/90F | GGATGATTGTGTAAACTGATTTCTC | 2383630/60F | ACATACAACTTCAGCTATTCTTGG |
| 2318272/74/90R | CATTCACCACCGTTCTTATGAC | 2383630/60R | GATTTCATAGCACGGTAGTTTGG |
| 939304F | TTTATTATAATCTATTGGTTGGTCGG | 954590F | GCGTAAGGGAAGTAGTTATCAG |
| 939304R | ATATTTATGTGATACGCAAAGGAG | 954590R | AATTCTGTTGCATTCAAATCAGG |
| 1016979F | TGATTATCGTCTTACCACAACTG | 1358230F | AAATCACCATATTGTGCTTCCC |
| 1016979R | TAAAGTAAGACAAAGCATACCCAC | 1358230R | GCATTCATTTATAGAACAAGTCCA |
| 2272936F | AAGCAGTGATGATATCTTTAGTTACC | 2678563F | TTACACATTTCTATGGAACGCT |
| 2272936R | TTTGAGACTCAAGAACGTTATCC | 2678563R | GGAAAGAGAAGCAATCAGACCT |
| D63bpF | ATTTATTCAGGAAGTAAAAGGC | D1200bpF | AGAACGATTGAAAGAATTACGAG |
| D63bpR | AAGTGTGCTGTATTCTAAAGTGC | D1200bpR | GGCGTTGCAGATACATACAC |
