# Supplementary material for: Genetic Variation in the Staphylococcus aureus 8325 Strain Lineage Revealed by Whole-Genome Sequencing
Source: PLoS One. 2013 Sep 30;8(9):e77122. doi: 10.1371/journal.pone.0077122 (PMC3786944; doi:10.1371/journal.pone.0077122)
Supplement: Table S2 — Deletions in 8325-4seq compared to NCTC8325. (PDF) [file pone.0077122.s004.pdf]

Table S2: Deletions in 8325-4 (seq) compared to NCTC8325

	Start	End	Size (bp)
Intergenic<br>SAOUHSC_00069 (*spa*)- SAOUHSC_00070 (*sarS*)	75276	75338	63
Prophage 12	1462575	1508604	46030
Prophage 11	1923408	1967018	43611
Prophage 13	2031924	2074647	42724
