# Supplementary material for: Genetic Variation in the Staphylococcus aureus 8325 Strain Lineage Revealed by Whole-Genome Sequencing
Source: PLoS One. 2013 Sep 30;8(9):e77122. doi: 10.1371/journal.pone.0077122 (PMC3786944; doi:10.1371/journal.pone.0077122)
Supplement: Table S3 — Oligonucleotide q-RT-PCR primers. (PDF) [file pone.0077122.s005.pdf]

Table S3: Oligonucleotide q-RT PCR primers

Primer	Sequence 5' -3'
sarS-F	CACCATAAATACCCTCAAACTGTTAGAG
sarS-R	TCATCTTCAGTTGAGCGTTCTTTT
sarS-P	AAAAAGCAAGGCTATCTAA
spa-F	CAGCAAACCATGCAGATGCTAA
spa-R	ACAGTTGTACCGATGAATGGATTTT
spa-P	AGCATTACCAGAAACT
16S-F	GATAGAGCCTTCCCCTTCGG
16S-R	CCGGCAGTCAACTTAGAGTGC
16S-P	ACATCTCACGACACGAGCTGACGACA
